# Physiological values of phagocytic capacity in marine mammals and alterations during pathological situations

**DOI:** 10.3389/fvets.2024.1389977

**Published:** 2024-05-02

**Authors:** Mar Felipo-Benavent, Alicia Martínez-Romero, Mónica Valls, Carlos Rojo-Solís, Teresa Álvaro, Daniel García-Párraga, Consuelo Rubio-Guerri, José-Enrique O’Connor

**Affiliations:** ^1^Laboratory of Cytomics, Joint Research Unit CIPF-UVEG, Department of Biochemistry and Molecular Biology, University of Valencia, Valencia, Spain; ^2^Department of Biomedical Sciences, Faculty of Health Sciences, Universidad CEU Cardenal Herrera, CEU Universities, Valencia, Spain; ^3^Cytomics Technological Service, Príncipe Felipe Research Center, Valencia, Spain; ^4^Veterinary Services, Avanqua Oceanográfic SL, Oceanogràfic, Ciudad de las Artes y las Ciencias, Valencia, Spain; ^5^Research Department, Fundación Oceanogràfic de la Comunitat Valenciana, Oceanogràfic, Ciudad de las Artes y las Ciencias, Valencia, Spain; ^6^Department of Pharmacy, Faculty of Health Sciences, Universidad CEU Cardenal Herrera, CEU Universities, Valencia, Spain; ^7^Laboratory of Cytomics, Department of Biochemistry and Molecular Biology, Faculty of Medicine, Valencia University, Valencia, Spain

**Keywords:** phagocytosis, marine mammals, respiratory burst, dolphins, beluga, walrus, sea lion, seal

## Abstract

The study of the immune function in marine mammals is essential to understand their physiology and can help to improve their welfare in the aquariums. Dedicating efforts to studying marine mammal physiology, pathophysiology, and implementing new diagnostic and therapeutic tools promote progress towards preventive medicine in aquariums by facilitating early detection and treatment of diseases. However, biological and clinical research on marine mammals is currently very limited due to difficult access to these species and their biological samples. With this objective, our group has adapted to marine mammals a commercially available assay routinely used to evaluate the phagocytic capacity of monocytes and granulocytes in human whole blood samples. We adapted IngoflowEx kit to bottlenose dolphins (*Tursiops truncatus*), beluga whales (*Delphinapterus leucas*), walruses (*Odobenus rosmarus*), Patagonian sea lions (*Otaria flavescens*), and harbor (*Phoca vitulina*). In this paper, we report the modifications carried out on the original protocol for their correct functioning in marine mammals. We obtained physiological values of phagocytic capacity in each species after repeated sampling for 4 years in various individuals of each species. Specific results revealed that the % phagocytic cells that ingested E.coli in bottlenose dolphins were 59.6 ± 1.27, in walruses 62.6 ± 2.17, in sea lions 57.5 ± 4.3, and in beluga whales 61.7 ± 1.4. In the case of the % phagocytic cells producing respiratory burst in bottlenose dolphins were 34.2 ± 3.6, in walruses 36.3 ± 4.3, in sea lions 40.8 ± 10.2, and in beluga whales 26.3 ± 3.7. These preliminary results can be used as a reference to detect alterations in phagocytic capacity either by immunosuppression or by exacerbation of the response in infectious inflammatory processes. Clinical applicability of the assay was verified in two clinical cases in which Ingoflow was useful to detect immune alterations in two diseased individuals, before and after the onset of clinical signs.

## 1 Introduction

Marine mammals, positioned at the apex of the aquatic food chain, are characterized by their long-life spans, and serve as the ultimate recipients of impacts within marine ecosystems (1, 2). These impacts can arise from various threats, including pollution, pathogens, and accidental fishing.

Presently, the discharge of pollutants into the ocean stands as a prominent global environmental challenge. Urban, agricultural, and industrial waste, totaling nearly 400 million tons annually, introduces a variety of heavy metals, solvents, and xenobiotics into marine ecosystems. Alarmingly, approximately 80% of the wastewater released into the sea remains untreated (3). These pollutants settle in the water and sediment on the ocean floor, where smaller organisms ingest them. Larger species then bioaccumulate contaminants through their diet (2). The biomagnification process leads predators to accumulate higher concentrations than their prey, given their extended exposure and longevity (4). Consequently, marine mammals serve as bioindicators in aquatic ecosystems, playing a crucial role in pathophysiological and toxicological studies (2). Various pollutants, such as heavy metals, insect repellents, pesticides, per- and polyfluoroalkyls (PFAs), polychlorinated biphenyls (PCBs), organochlorine pesticides (OCPs) or tributyltins, have been identified in tissues of different marine mammal species. Specifically, striped dolphins (*Stenella coeruleoalba*) (5, 6), Fraser’s dolphins (*Lagenodelphis hosei*) (7), common dolphins (*Delphinus delphis*) (8), killer whales (*Orcinus orca*), polar bears (*Ursus maritimus*) (9) or harbour seal (*Phoca vitulina*) (10) presented one or more of these compounds. In addition, by 2014, the worldwide production of plastics had exceeded 300 million metric tons annually. Plastic waste has been identified across all major marine environments globally, ranging in size from microns to meters (11). This pervasive presence of plastics is a growing concern, particularly as marine mammals are also known to suffer from the impacts of plastic pollution. In fact other studies confirmed the presence of phthalates in the urine of bottlenose dolphins (*Tursiops truncatus*), demonstrating their dietary exposure to microplastics or plastic derivates (12).

The exposure to certain contaminants, such as PCBs, pesticides, or heavy metals, can lead to diseases in animals. Specifically, the direct relationship between exposure to certain xenobiotics and alterations in immune functionality has been demonstrated in several *in vitro* studies conducted in different marine mammal species. On one hand, certain PCBs have been shown to decrease the function of granulocytes and lymphocytes in killer whales and polar bears (9). PCBs have also been associated with a reduction in NK cell activity in Californian sea lions (*Zalophus californianus*) (13). On the other hand, OCPs have been found to reduce the phagocytic capacity in killer whales (9). Heavy metals such as Hg and Cd can alter the viability and function of lymphocytes and granulocytes in bottlenose dolphins (14). Also, tributyltin and some of its metabolites, especially dibutyltin, have the potential to alter leukocyte function in seals, leading to a reduction in the activity of phagocytes, B lymphocytes, and NK cells (10). Additionally, silver nanoparticles from textile and cosmetic industries have been observed to generate a decreased proliferative capacity of lymphocytes and induce apoptosis in dolphins (15).

Nevertheless, the human impact on the marine ecosystem is not limited to the leakage of pollutants. Overfishing has resulted in competition for fish between humans and cetaceans (16), leading to increased accidental net captures (17). Furthermore, maritime traffic, military maneuvers, and oil or gas extraction generate high levels of sound in the ocean (18), often overlapping or surpassing the sounds emitted by marine mammals for communication, reproduction, or hunting (19). All these situations can trigger an acute stress response in the animals, accompanied by alterations in behavior and the release of cortisol and catecholamines into the bloodstream (18). Catecholamines, such as epinephrine and norepinephrine, increase respiratory and heart rates and the arrival of blood to the muscles, facilitating the fight or flight response (20). This occurs at the expense of the function of other structures, such as the digestive, reproductive, or immune systems. Both catecholamines and glucocorticoids affect the functionality of immune cells by binding to specific leukocyte receptors, triggering alterations in cytokine production and inhibition of cell maturation and mobilization. The primary stress molecule in mammals is cortisol, which binds to glucocorticoid type II receptors in the cytoplasm of immune cells. Subsequently, this receptor is translocated to the cell nucleus, where it alters the production of numerous cytokines such as interferon-gamma (IFNγ), interleukins IL-1, IL-2, and IL-6, or tumor necrosis factor (TNFα) (20). Therefore, stress can also impair immune function in marine mammals.

Additionally, marine mammals are exposed to numerous pathogens in the ocean. Some of them can enter through untreated human wastewater that is discharged into the sea (3), while others are natural environmental organisms. As is well known, bacterial or fungal infections (21, 22), may exacerbate immune function, while others, typically of viral origin, may induce immunosuppression in animals.

Finally, as is widely recognized, stress can induce significant physiological alterations in living organisms, one of which, in the animal kingdom, is immunosuppression (23). It is well known that animals under human care can suffer stress in specific conditions and this result in a decrease of the functionality of the immune system (24).

In summary, the immune function of marine mammals can be altered through various mechanisms and serves as a biomarker not only of the health status of the animal but also of the environment in which it resides. Establishing standardizable methods to monitor immune function in these species is necessary to determine, firstly, the physiological immune functionality in healthy animals, and secondly, immune alterations due to pathologies, stress, or exposure to xenobiotics. This is particularly relevant in aquarium veterinary clinics, as these species may not exhibit signs of weakness until the disease has advanced, to avoid appearing vulnerable to larger predators in the wild. Routinely measuring immune function could be a valuable tool in the early diagnosis and monitoring of certain pathologies in marine mammals.

In this study, our group concentrates on adapting a human commercial assay for measuring the phagocytic capacity of granulocytes and monocytes to marine mammals. The objective was to establish a fast, simple, and cell-respectful methodology that can be employed to assess the phagocytosis of animals, either to evaluate their health status or to conduct immunity studies. The use of commercial solutions ensures a high level of standardization in experiments, facilitating the comparison of results across studies conducted by different research groups or aquariums. Specifically, our focus has been on adapting the IngoFlow assay for use in bottlenose dolphins (*Tursiops truncatus*), beluga whales (*Delphinapterus leucas*), walruses (*Odobenus rosmarus*), seals (*Phoca vitulina)* and sea lions (*Otaria flavescens*). The assay measures pathogen ingestion and our group simultaneously analyzed respiratory burst in granulocytes and monocytes from whole blood samples. Subsequently, we have defined a range of physiological values for each parameter in each species, considering the limited sample size and the living conditions of the individuals. We have observed alterations in the phagocytic capacity of animals with various infections, demonstrating the diagnostic and clinical effectiveness of the method.

## 2 Materials and methods

### 2.1 Blood sampling

Heparinized whole blood was obtained from healthy animals under human care at Oceanogràfic Aquarium (Valencia, Spain). One mL of venous blood was drawn from the ventral face of the caudal fin in cetaceans and from interdigital veins on the caudal flippers in pinnipeds. For that, 21G gauge size Butterfly needle (Venofix^®^ by Fa. Braun) and 10 mL single-use syringes (Covetrus) were used. All the animals had been previously trained for blood collection. Samples were analyzed in the Cytomics Laboratory at the Príncipe Felipe Research Center (CIPF, Valencia, Spain) within 2 h after being obtained. We obtained 44 samples from 15 bottlenose dolphins (*Tursiops truncatus*), 20 samples from 3 beluga whales (*Delphinapterus leucas*), 18 samples from 3 walruses (*Odobenus Rosmarus*), 4 samples from 4 sea lions (*Otaria flavescens*) and 2 samples from 1 seal (*Phoca vitulina*). All the experiments were approved by the Animal Care Committee of the Oceanogràfic Aquarium (Reference: OCE-6-17).

### 2.2 Reactive and solutions

#### 2.2.1 Fluorochromes

Hydroethidine (HE) is an indicator of intracellular superoxide. It was prepared at 1 mg/mL in dimethyl sulphoxide (DMSO) and used at a final concentration of 0.3 μg/mL. Hoechst 33342 (HO) is a vital nuclear marker. It was prepared at 1 mg/mL in distilled H_2_O and used at a final concentration of 3 μg/mL. Propidium iodide (PI) is a cell viability indicator that stains cells with damaged membrane. It was prepared at 1 mg/mL in distilled H_2_O, and used at a final concentration of 5 μg/mL. All fluorochromes were obtained from Merck (Darmstadt, Germany).

#### 2.2.2 IngoFlowEx Kit for quantification of phagocytic activity

IngoFlowEx (phagocytic assay) (Exbio, Prague, Czech Republic, Cat. ED7040) was adapted to evaluate the phagocytic capacity of monocytes and granulocytes in marine mammals. This kit includes: suspension of fluorescein-labelled *E. coli* bacteria (*E. coli*-FITC), quenching solution (trypan blue), solution for erythrocyte lysis and leukocyte fixation, washing buffer and DNA staining solution (PI). Trypan blue (TB) is introduced to freely diffuse into the cell, ensuring uniform distribution throughout the cytoplasm and nucleus. TB can swiftly diffuse through cellular and nuclear membranes. At suitable concentrations, this indiscriminate dispersion of dye molecules positions them effectively in proximity and orientation to autofluorescent or nonspecifically bound fluorescent molecules, enabling quenching to take place (25).

#### 2.2.3 Reactive

Cytochalasin A (ENZO, Farmingdale, NY, United States) is a phagocytosis inhibitor. It was prepared at 10 mM in DMSO and aliquoted and stored at −20°C. It was used at a final concentration of 400 μM.

#### 2.2.4 Buffers, solutions and mediums

Versalyse Lysing Solution (Beckman Coulter, Brea, CA, United States) was used as a non-fixative erythrocyte lysant. RPMI 1640 medium (GIBCO, 21875034) was used to reconstitute the samples. PBS phosphate buffer saline pH 7.4 (Gibco-Thermo Fisher, Waltham, MA, United States) was used as a general buffer.

### 2.3 Quantification of phagocytic activity

To assess phagocytic cells function, we studied two essential phases in the phagocytosis process: pathogen ingestion and pathogen destruction by the respiratory burst. The phagocytic assay measures the pathogen ingestion capacity of monocytes and granulocytes in humans. Our group, which was routinely using this kit at that time to measure immune function in humans, introduced several modifications of the original experimental design to optimize its application to marine mammals and added the simultaneous measurement of respiratory burst using HE. The green fluorescent *E. coli* bacteria provided by the phagocytic assay were used as stimulus, since, like in humans, it is also capable of infecting cetaceans and pinnipeds (21, 22).

#### 2.3.1 Phagocytic assay principle

This assay is based on measuring the fluorescence of phagocytic cells after ingesting green, fluorescent *E. coli*-FITC. Bacteria is added to the blood and incubated at 37°C (physiological temperature of marine mammals) for 30 min. In parallel, the negative control, not exposed to the pathogen, is incubated too and used to establish in the flow cytometer (FC) the limit between the cells that have phagocytosed the bacteria and those that have not. At the end of the incubation, the sample may contain: (a) autofluorescent phagocytic cells that have not ingested *E. coli*-FITC. (b) Green-fluorescent phagocytic cells with *E. coli*-FITC attached to the plasma membrane but not yet ingested. (c) Green-fluorescent phagocytic cells that have actually ingested *E. coli*-FITC. (d) Green-fluorescent free bacteria.(e) The rest of the blood components (erythrocytes, platelets, debris…).

Erythrocytes are lysed and cell debris and uningested bacteria are removed by washing with PBS. In addition, leukocytes are fixed and stained with PI for being identified as nucleated cells in the flow cytometer. The fluorescence of bacteria attached to the cell surface but not yet ingested is quenched by trypan blue.

#### 2.3.2 Modifications of the assay for its application to marine mammals

We have improved the biological relevance of the original assay by incorporating additional reagents and discarding others provided by the kit. First, HE, an indicator of superoxide radical generated during the oxidative burst is incorporated to the assay. After oxidation during the respiratory burst, HE yields ethidium that intercalates into DNA and emits orange-red fluorescence. So, the phagocytic capacity (estimated by the green fluorescence of the bacteria) and the respiratory burst production (estimated by the red fluorescence of the HE) can be simultaneously evaluated. Since the fluorescence properties of HE-generated ethidium overlap those of PI, we replaced this dye by HO to discriminate nucleated cells.

On the other hand, we have introduced several modifications of the original experimental design to optimize its application to marine mammals, as listed below:

- Including an additional negative biological control:

The negative control proposed by the manufacturer reports the cell autofluorescence. An additional biological negative control was included by using cytochalasin A as phagocytosis inhibitor. This control is more comparable to the test tube, since it contains the same components (*E. coli-*FITC and HE), but avoiding the phagocytosis process.

- Increased incubation period:

We have extended the incubation period from 30 min (according to the manufacturer) to 1 h at 37°C, observing better results in the discrimination between the population of phagocytic cells that had ingested the bacteria and those that had not. The temperature was set to 37°C because the physiological temperature of marine mammals is around 37°C, as it is in humans. Thus, we are recreating the same physiological conditions that would be encountered *in vivo.*

- Changing to a non-fixing erythrocyte lysis solution:

The erythrocyte lysis solution provided by phagocytic assay is also fixative. To keep the cells alive, we replaced it with VersaLyse Lysing Solution (Beckman Coulter, California, United States), a non-fixing solution. VersaLyse Lysing Solution is a reagent used to lyse red blood cells from any biological fluid and, in particular, to lyse erythrocytes from whole blood. It is a highly specific and very gentle lysing solution that ruptures erythrocytes but, as it does not fix all cells, does not affect the viability of the remaining immune cells. In the cetacean samples, the manufacturer’s instructions were followed observing good results, however pinniped samples required twice the volume of VersaLyse or the incubation time with the lysis solution to achieve similar results. The variation in the impact of the lysis solution is attributed to the elevated blood lipid content present in pinnipeds. This may hinder, to some extent, the interaction between the reagent and erythrocytes, thereby impeding its effectiveness. This heightened blood lipid content has been documented in previous studies (26).

- Using RPMI medium to preserve cells until cytometric analysis:

The original assay uses PBS to preserve cells until the analysis. To be more respectful with cell viability, PBS was replaced with warm RPMI medium.

- Trypan blue addition time:

In the original assay trypan blue is added after the incubation with *E. coli*-FITC. We observed better results quenching the fluorescence of non-ingested bacteria by adding it just before the reading in the cytometer, avoiding washing after its addition and ensuring its permanence in the sample to quench the fluorescence of free or membrane attached bacteria.

#### 2.3.3 Protocol for the simultaneous measurement of the ingestion capacity and respiratory burst production of monocytes and granulocytes in marine mammals

Five tubes were prepared per analysis: (1) *Negative control*: to evaluate the autofluorescence of the sample. Without *E. coli*-FITC and HE. (2) *Control of cellular basal oxidative state*: adding HE but not *E. coli*-FITC. This tube allows also for fluorescence compensation of HE into FITC. (3) *Biological negative control*: inhibiting phagocytosis with cytochalasin A. Adding *E. coli*-FITC and HE. (4) *Control of phagocytosis*: adding *E. coli*-FITC but not HE. This tube allows also for fluorescence compensation of FITC into HE. (5) *Test tube*: adding *E. coli*-FITC and HE for the simultaneous measurement of pathogen ingestion and respiratory burst production capacities.

**Figure 1:**
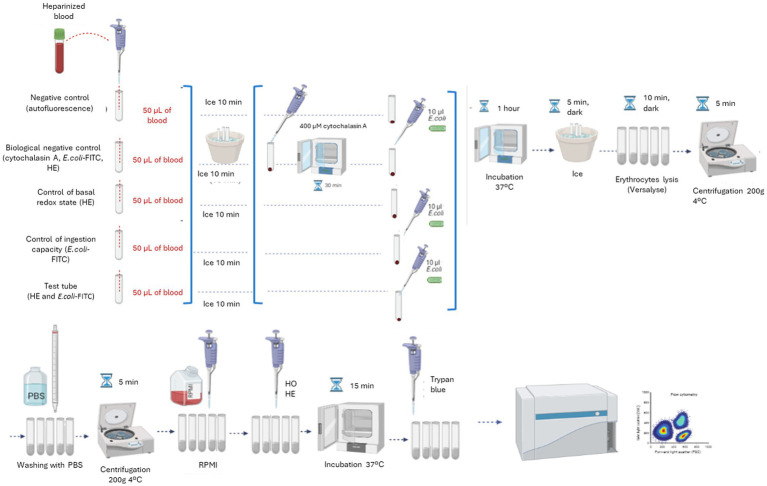
Diagram of the definitive protocol steps for the simultaneous measurement of the ingestion capacity and the respiratory burst production of monocytes and granulocytes in marine mammals.

The protocol steps are shown in Figure 1. First, 50 μL of heparinized blood are added to each tube. The tubes are kept on ice for 10 min. After this time, the biological negative control is preincubated with cytochalasin A at a final concentration of 400 μM for 30 min at 37°C CO_2_ in an incubator. Subsequently, 10 μL of *E. coli*-FITC are added to the biological and ingestion controls and to the experimental tube. All tubes were incubated for 1 h at 37°C in the CO_2_ incubator. After incubation, the tubes were placed on ice and in dark for 5 min to stop the phagocytosis process. Then, Versalyse Lysing Solution is added to each tube in cetacean samples (500 μL) or 1 mL in the pinniped samples (1 mL). The lysis solution is allowed to act for 10 min at room temperature and in the dark. Once lysed, the samples are centrifuged for 5 min at 200 g and 4°C. The supernatant is removed, and samples washed twice with 3 mL of cold filtered PBS. After removing the supernatant, the samples are resuspended in 300 μL of tempered RPMI. Finally, HO and HE are added to the corresponding tubes at a final concentration of 3 μg/mL and 0.3 μg/mL, respectively, and incubated for 15 min at 37°C in a CO_2_ incubator. Before the analysis in the flow cytometer, 10 μL of trypan blue solution is added to each tube. Samples are analyzed on the cytometer immediately.

#### 2.3.4 Flow cytometer settings

The CytoFlex S flow cytometer with blue (488 nm) and violet (405 nm) lasers (Beckman Coulter, Brea, CA, United States) was configured to collect forward scatter (FSC) and side scatter (SSC) signals as well as the fluorescence emitted by the bacteria *E. coli*-FITC (exc 488 nm/em 525 nm, FITC channel), HO (exc 405 nm/em 450 nm, BP450 channel) and HE (exc 561 nm/em 585 nm, PE channel).

**Figure 2:**
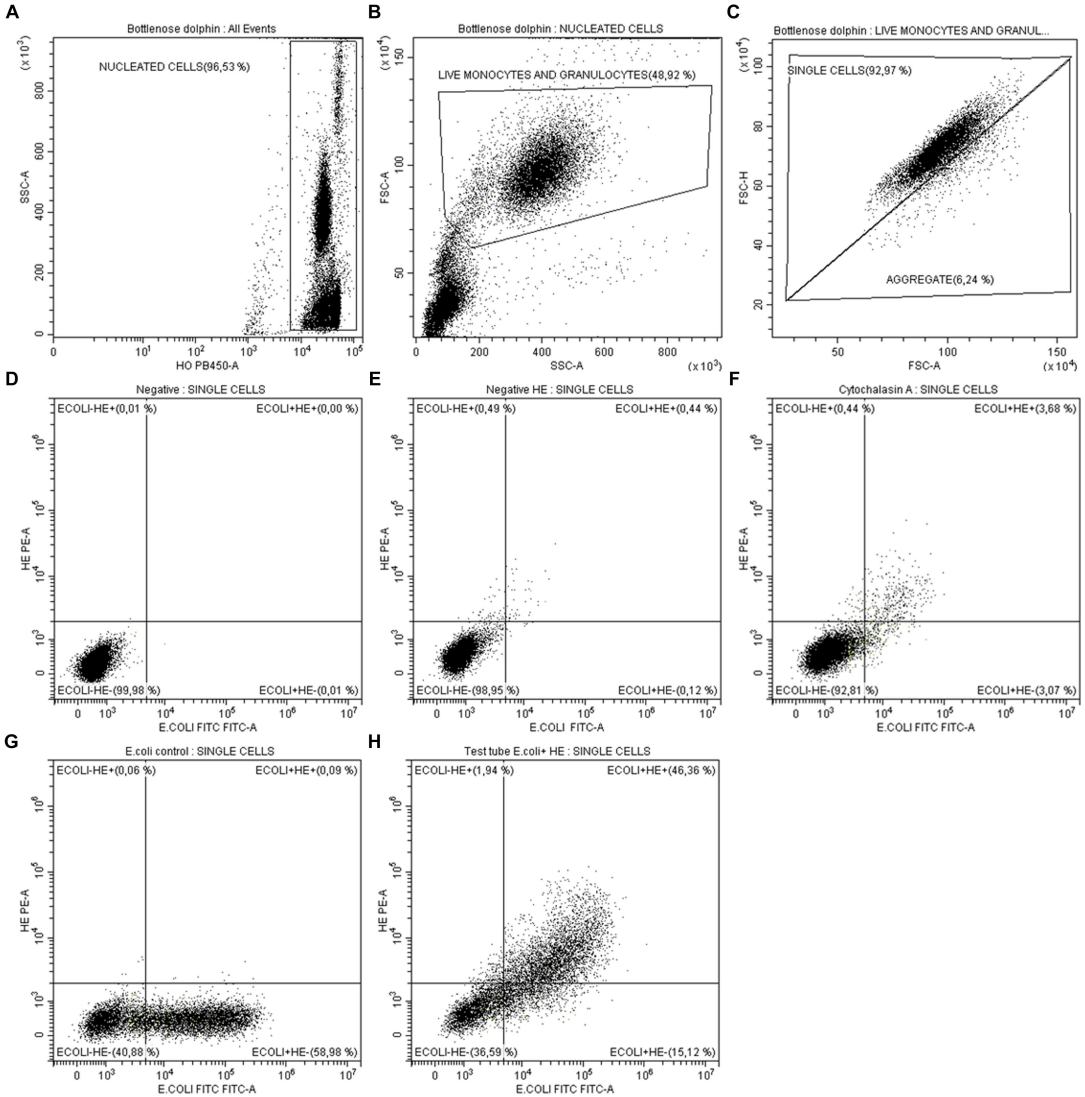
Analysis by flow cytometry of monocytes and granulocytes phagocytic capacity in marine mammals. **(A)** Identification and gating of nucleated cells as HO^+^ events (leukocytes). **(B)** From leukocytes, gating of live monocytes and granulocytes by morphology (FSC-SSC). **(C)** Aggregates exclusion by analysis of FSC-height vs. FSC-area of live monocytes and granulocytes. To ensure the same number of events in all the experiments carried out, a limit of 10,000 events was established in the single cell population. From single events: **(D)** Negative tube: since *E. coli*-FITC and HE-PE were not added, all events are negative for both fluorescences. **(E)** Control of cells basal redox state: since only was added HE. **(F)** Biological negative control: few events present *E. coli*-FITC and HE-PE fluorescence after inhibiting phagocytosis with cytochalasin A. **(G)** Control of ingestion capacity: percentage of cells present *E. coli*-FITC fluorescence after having ingested it. HE was not added. **(H)** Test tube: shows the percentage of cells that have not ingested *E. coli*-FITC and produced respiratory burst (*E. coli*^−^ HE^−^), the percentage of cells that ingested *E. coli*-FITC but have not yet produced respiratory burst (*E. coli*^+^ HE^−^) and the percentage of cells that ingested *E. coli*-FITC and produced respiratory burst (*E. coli*^+^ HE^+^).

The population identification and gating strategy is detailed in Figure 2. Leukocytes were identified as HO fluorescence positive events (Figure 2A). From leukocytes, phagocytic cells (monocytes and granulocytes) were selected by morphology, according to their relative size (FSC) and internal complexity (SSC). Dead cells were also discarded morphologically in this step (Figure 2B). Subsequently, cell aggregates were electronically removed using the area (FSC-A) and peak (FSC-H) signals of the FSC (Figure 2C). To ensure the same number of events in all the experiments carried out, a limit of 10,000 events was established in the single cell population in all the analyzes carried out. In live and individual monocytes and granulocytes, the phagocytosis capacity and respiratory burst production were quantified simultaneously. For this, a dot-plot comparing the *E. coli*-FITC and HE-PE signals was used in the different tubes: (1) *Negative control*: in this tube phagocytic cells were not exposed to either *E. coli*-FITC or HE, so all events are negative to both fluorescence, giving the cells autofluorescence (Figure 2D). (2) *Control of the basal oxidative state*: provides an estimation of the cells basal redox state, since HE is added but not *E. coli*-FITC (Figure 2E). (3) *Biological negative control*: in this tube the phagocytosis is inhibited with cytochalasin A, so few events present FITC and HE fluorescence (Figure 2F). (4) *Control of ingestion capacity*: in this tube *E. coli*-FITC is added but not HE, observing the percentage of phagocytic cells emitting bacteria-dependent fluorescence (Figure 2G). (5) *Test tube:* this tube shows the percentage of phagocytic cells that: have not ingested and destroyed the bacteria (*E. coli*^−^ HE^−^); contain bacteria inside but have not yet destroyed them (*E. coli*^+^ HE^−^); have ingested bacteria and are producing the respiratory burst (*E. coli*^+^ HE^+^) (Figure 2H).

#### 2.3.5 Numerical analysis of phagocytic capacity

Two parameters were assessed using Flowjo software: (a) *The percentage of cells that ingested the pathogen:* calculated by subtracting the percentage of FITC positive events of the biological control from that of the experimental tube. (b) *The percentage of cells generating the respiratory burst after ingesting the bacteria:* calculated by subtracting the percentage of *E. coli*^+^/HE^+^ cells of biological control from that of the experimental tube.

### 2.4 Statistical analysis

The mean, median and standard error of the median (SEM) were calculated for each parameter in all the species. To evaluate differences between animals per sex and age we performed *t*-test in GraphPad Prism 5. The results were considered statistically significant when *p*-value <0.05. To evaluate differences between species, the distribution of the data for each parameter was tested using Shapiro–Wilk test. In all cases the data distribution was normal one-way ANOVA test was utilized.

## 3 Results

### 3.1 Adapting a standardized assay to measure phagocytic capacity in marine mammals

It is the first time that a commercial kit intended for human medicine has been adapted to measure phagocytic capacity in dolphins, beluga whales, walruses, sea lions and seals. It represents a novelty in the field of marine mammal immunology and a new study tool that allows the standardization of the results in different investigations. Since IngoFlow Kit is originally intended for diagnosis in human medicine, it undergoes numerous and exhaustive quality controls before being commercialized to hospitals or research centers. So, the use of this adapted kit with added solutions and modified protocol implies the standardization of the results between individuals, samples from the same individual at different times or even between studies. Adapting this assay to marine mammals allows the results obtained between different research groups to be very reliably comparable.

On the other hand, the use of HE to study the respiratory burst production is also an innovation in immunological studies in marine mammals, demonstrating its usefulness as superoxide marker in these species.

### 3.2 Obtaining physiological values of phagocytic capacity in healthy marine mammals

In order to detect eventual alterations in phagocytic capacity, first it was necessary a previous descriptive study of phagocytes normal function for each specie. With this purpose, periodically samples of individuals of different species were analyzed over 4 years. Specifically, 44 total samples were obtained from 15 dolphins, 20 samples from 3 belugas, 18 samples from three walruses, and to a lesser extent, 4 samples from 4 sea lions and 2 samples from a seal. In the last two species the data must be taken with caution due to the small number of samples, but it can be indicative for future studies. The median for each animal was obtained, discarding the data obtained when the animal was sick. Then, the mean and standard error (SEM) of the specie was calculated obtaining a range of physiological values for each parameter in healthy animals of the different species.

The most robust data were obtained in dolphins, since the population size and the number of samples per individual were greater. This methodology was useful for the internal control of the animals under study, but to extrapolate the results to other animals it would be convenient to carry out routine analysis in animals housed in different aquariums.

#### 3.2.1 Ranges of phagocytic capacity of monocytes and granulocytes in marine mammals

The ranges of the physiological values for ingestion capacity and respiratory burst production in each species, and the effects of sex and age are detailed below (Tables 1–4). The physiological values were calculated for all species except seals, since in that case we only had samples from one animal. In this case, the main objective was to set up the methodology for its use in future studies. On the other hand, the results obtained in sea lions should be taken with caution due to the small sample size.

**Table 1:** Physiological phagocytic capacity values in dolphins after 1 h of incubation with the pathogen.

	% phagocytic cells that ingested *E. coli* (mean ± SEM)	% phagocytic cells producing respiratory burst (mean ± SEM)
Bottlenose dolphins *n* = 15, 44 samples	59.6 ± 1.27 (min: 53; max: 69)	34.2 ± 3.6 (min: 9; max: 48.6)
Adults *n* = 12, 36 samples	59.1 ± 1.3	32 ± 3.8
Calves *n* = 3, 8 samples	61.9 ± 3.5	48.3 ± 0.3
Males *n* = 5, 17 samples	60.3 ± 2.7	42.4 ± 2.15
Females *n* = 10, 27 samples	59.3 ± 1.5	31 ± 4.7

Forty-four samples from 15 dolphins were analyzed. The table shows the % of monocytes and granulocytes capable of ingest the pathogen and produce respiratory burst in the global population and by sex and age. The minimum and maximum values are also detailed. No significant differences were observed by sex or age (NSD: *p* ≥ 0.05).

**Table 2:** Physiological phagocytic capacity values in beluga whales after 1 h of incubation with the pathogen.

	% phagocytic cells that ingested *E. coli* (mean ± SEM)	% phagocytic cells producing respiratory burst (mean ± SEM)
Walruses *n* = 3, 18 samples (6 per animal)	62.6 ± 2.17 (min: 50; max: 79)	36.3 ± 4.3 (min: 13; max: 64)

Eighteen samples from 3 walruses were analyzed. The table shows the % of monocytes and granulocytes capable of ingest the pathogen and produce respiratory burst in the global population. All animals were adult females so differences by sex or age could not be evaluated. No significant differences were observed between the animals (NSD: *p* ≥ 0.05). The minimum and maximum values are also detailed in the table.

**Table 3:** Physiological phagocytic capacity values in walruses after 1 h of incubation with the pathogen.

	% phagocytic cells that ingested *E. coli* (mean ± SEM)	% phagocytic cells producing respiratory burst (mean ± SEM)
Beluga whales *n* = 3, 20 samples	61.7 ± 1.4 (min: 52; max: 74)	26.3 ± 3.7 (min: 5.8; max: 57.2)
Adults *n* = 2, 14 samples	63.3 ± 1.8	29.7 ± 4.4
Calf *n* = 1, 6 samples	58 ± 0.9	18.6 ± 6.2
Males *n* = 2, 12 samples	58.7 ± 0.75	22.6 ± 4.5
Female *n* = 1, 8 samples	65 ± 2.4	32 ± 6.3

Twenty samples from 3 beluga whales were analyzed. The table shows the % of monocytes and granulocytes capable of ingest the pathogen and produce respiratory burst in the global population and by sex and age. The minimum and maximum values are also detailed. No significant differences were observed by age (NSD: *p* ≥ 0.05). Phagocytic cells from female presented a significant higher ingestion capacity than males (*p* = 0.0175*).

**Table 4:** Physiological phagocytic capacity values in sea lions after 1 h of incubation with the pathogen.

	% phagocytic cells that ingested *E. coli* (mean ± SEM)	% phagocytic cells producing respiratory burst (mean ± SEM)
Sea lions *n* = 4, 4 samples	57.5 ± 4.3 (min: 51; max: 70)	40.8 ± 10.2 (min: 21; max: 68)
Males *n* = 2, 2 samples	54.6 ± 2.5	32.15 ± 11.25
Females *n* = 2, 2 samples	60.5 ± 9.5	49.5 ± 18.5

Four samples from 4 sea lions were analyzed. The table shows the % of monocytes and granulocytes capable of ingest the pathogen and produce respiratory burst in the global population and by sex. All the animals were adults so differences by age could not be evaluated. The minimum and maximum values are also detailed. No significant differences were observed by sex (NSD: *p* ≥ 0.05). These results should be taken with caution due to the small sample size.

##### 3.2.1.1 Bottlenose dolphins

The physiological values of phagocytic function obtained from more than 40 samples from 15 dolphins are detailed in Table 1. No significant differences were observed between animals of different ages or sexes.

##### 3.2.1.2 Beluga whales

The physiological values of phagocytic function obtained from 20 samples from 3 beluga whales are detailed in Table 2. No significant differences were observed between animals of different ages, however, the female presented a significantly higher percentage of phagocytic cells capable of ingesting the pathogen than males (*p* = 0.0175*). Since we only provided samples from one female, this finding could be due to intrinsic characteristics of the individual. To confirm the dependence on the gender of this effect, it is necessary to increase the population size.

##### 3.2.1.3 Walruses

The physiological values of phagocytic function obtained from 18 samples from 3 walruses are detailed in Table 3. As all the walruses were females and of the same age, no sex or age differences could be investigated in this species.

##### 3.2.1.4 Sea lions

For the study, 4 samples from 4 healthy adult sea lions were obtained. Although the sample size is insufficient to obtain physiological values of the population, Table 4 shows the results obtained in this group, which can be indicative of the species. It would be necessary to expand the sample size in future studies. No statistically significant differences were observed between sexes.

#### 3.2.2 Differences in phagocytic capacity among marine mammal species

Shapiro Wilks test determined the normal distribution of the data for both parameters studied: pathogen ingestion capacity and respiratory burst production, presenting the *p*-values *p* = 0.23 and *p* = 0.09, respectively, (*p* > 0.05). No statistically significant differences were observed between the different species in pathogen ingestion capacity (*p* = 0.2) and respiratory burst production (*p* = 0.87) using one way ANOVA.

### 3.3 Application of phagocytic capacity measurement to health monitoring in clinical cases

After establishing the physiological phagocytic capacity values per species, it was possible to detect alterations in several clinical cases of animals with different disorders.

#### 3.3.1 Clinical case 1: bottlenose dolphin with fungal infection

One dolphin in the aquarium suffered chronic intermittent respiratory infections due to the fungus *Rhizopus microsporus*. This fungus belongs to the class Zygomycetes, which are widely distributed in the environment. The most common mode of infection is by inhalation of spores traveling in the air. The first defensive barriers against zygomycetes are the mucous membranes and the endothelium, although some spores can invade it. Phagocytic cells act against the fungus, preventing its germination and the proliferation of hyphae (27). Normally, the spores are easily fought by the host’s phagocytic cells, however, if the spore load is very high or the animal is immunodeppressed, hyphae are developed producing lesions in the area (27).

**Figure 3:**
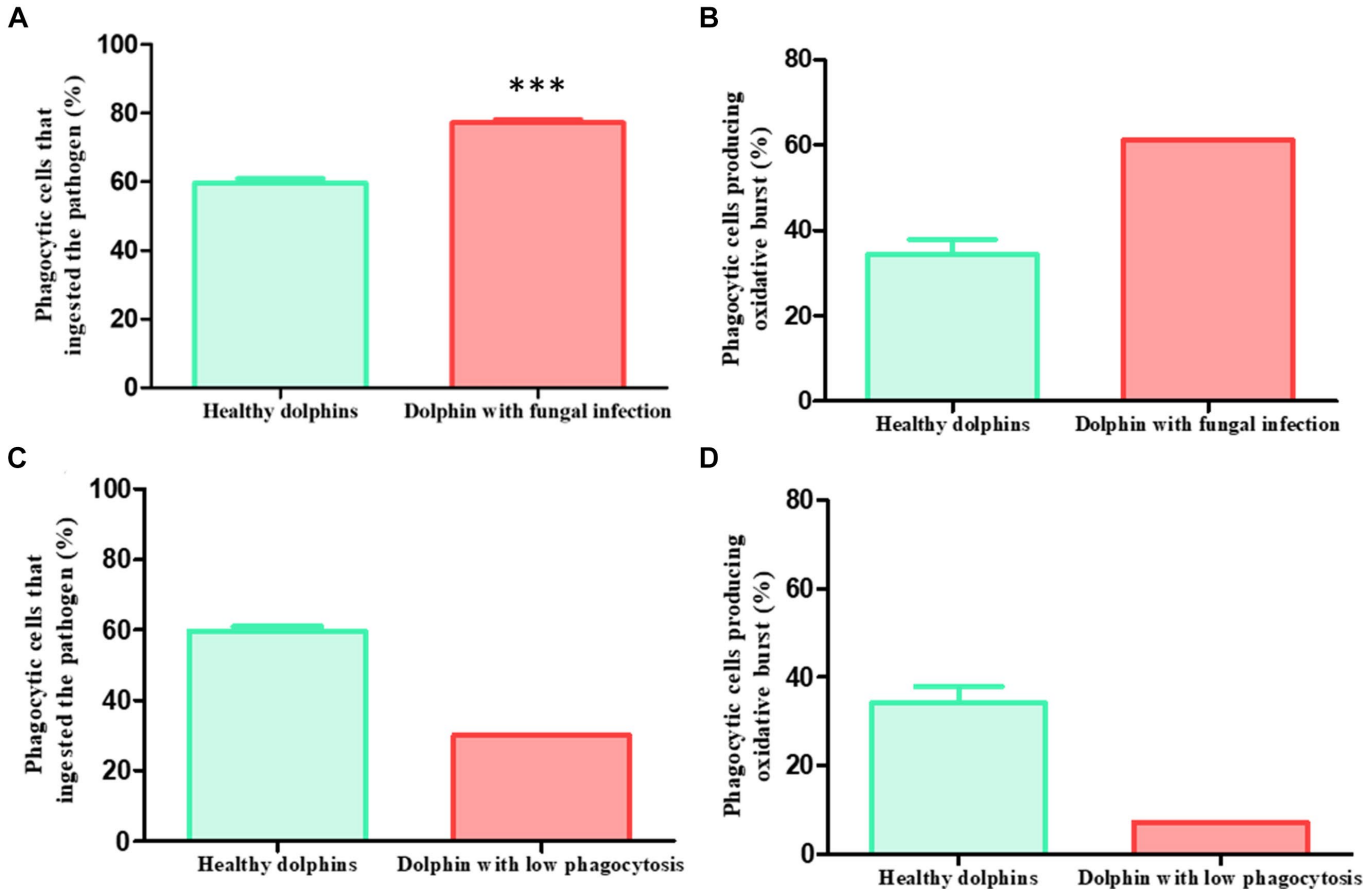
Clinical cases. Phagocytic capacity values in two sick dolphins. Clinical case 1: **(A)** dolphin with recurrent fungal infection presented pathogen ingestion capacity values significantly higher than healthy average in three different episodes (*p* < 0.0001***) (sick: 79, 76.6 and 76%, mean ± SEM: 77.2 ± 0.9%; healthy: 59.6 ± 1.27%). **(B)** During one episode, it also presented a higher capacity to generate respiratory burst than the healthy average (sick: 61%; healthy: 34.2 ± 3.6%). Clinical case 2: 3 days before showing digestive symptoms due to yeast, a dolphin presented a lower phagocytic capacity than healthy average in a routine control. **(C)** This effect was observed both in the ingestion capacity (sick: 30%; healthy: 59.6 ± 1.27%) **(D)** and in the production of the respiratory burst (sick: 7.2%; healthy: 34.2 ± 3.6%).

The phagocytic capacity values of the dolphin during three different episodes of *Rhizopus microsporus* infection are detailed next. In all three cases, the animal showed a percentage of phagocytic cells ingesting the pathogen significantly higher than the population average. Specifically, the percentages were the following: episode 1: 79%; episode 2: 76.6%; episode 3: 76%. The mean ± SEM of the three episodes was 77.2 ± 0.9%, which is significantly higher (*p* < 0.0001***) than the mean presented by the population of healthy dolphins (59.6 ± 1.27%) (Figure 3A). Furthermore, during one of the episodes it also showed an increase in the percentage of cells that produced the respiratory burst (61%) compared to the healthy population (34.2 ± 3.6%) (Figure 3B).

These findings not only demonstrate the higher activation of the animal’s phagocytic cells during infection trying to combat the fungus. It also validates the usefulness of the assay to detect alterations in the immune function of animals during certain pathologies, supposing a new diagnostic tool in clinical veterinary medicine in the aquariums.

#### 3.3.2 Clinical case 2: bottlenose dolphin with low phagocytic capacity and subsequent yeast infection

During one of the routinary samplings of the animals, it was observed that a dolphin presented a percentage of phagocytic cells ingesting the pathogen (30%) and producing the respiratory burst (7.2%) lower than the average of the healthy population (59 0.6 ± 1.27% and 34.2 ± 3.6% respectively) (Figures 3C,D). The animal was apparently healthy and did not show any signs of illness. Three days later the dolphin showed digestive symptoms, mainly diarrhea. The veterinary staff diagnosed a yeast infection.

Some types of yeast are part of the natural intestinal flora of dolphins. Immunodepression has been described as one of the key factors in the imbalance of the intestinal flora, which can trigger, among other things, an overgrowth of yeast that is accompanied by digestive sickness (28).

This clinical case is of special relevance, since the finding of the diminished phagocytic capacity in the animal preceded the appearance of the symptoms, demonstrating the usefulness of this assay to early detect disorders related to alterations in immune function, contributing to the improvement of preventative aquarium medicine.

## 4 Discussion

Although phagocytosis has been previously studied in marine mammals, the assay adapted by our group in this work represents a significant advancement in the methodology. The main advantage lies in the standardization of the method that ensures its reliable and comparable use in different research groups or aquariums worldwide. In most prior studies, the methodologies used to measure phagocytosis and respiratory burst in marine mammals relied either on the use of *Staphylococcus aureus* with prelabelled PI (1, 15, 29) or of fluorescent polystyrene beads (10, 14, 30–32) as a stimulus. In the first case, the staining of the pathogen is homemade, making it challenging to compare results between studies. In the second case, the stimulus used is not biological, deviating from the natural response of phagocytes. In our assay, we employed green, fluorescent *E. coli*, a natural pathogen of marine mammals (21, 22), as a biological stimulus. Additionally, the bacteria were prepared by the commercial company Exbio, following rigorous quality and standardization controls to ensure identical and comparable use in all the kits sold. This assay is also more respectful to the cells compared to those conducted in previous studies, where leukocytes were isolated, followed by cell counts before incubation with the pathogen, erythrocyte lysis, and cell fixation with 1% paraformaldehyde (1, 15, 29, 32). The excessive manipulation of leukocytes can alter their functionality. In our case, whole blood was used to closely mimic the *in vivo* physiological immune response, keeping the sample as unaltered as possible during incubation with bacteria. Erythrocyte lysis and washes are performed in the final steps, ensuring optimal function of the cells during the phagocytosis process. Respiratory burst has been measured previously in different species of marine mammals, either simultaneously with phagocytosis (1, 15) or separately (29). For this purpose, the reagent dichlorofluorescein diacetate (DCFH-DA) has been used (1, 15, 32). Our choice of HE was based on preventing fluorescence overlap of the bacterium and the oxidative burst indicator. Thus, our assay with the IngoFlow Kit is a new and useful tool to compare results between studies, while also being more respectful of cell viability and functionality. The adaptation of the IngoFlowEx assay to marine mammals enables its use as a diagnostic assay in the daily veterinary clinic of aquariums or as a new tool in future immune studies.

Phagocytosis and respiratory burst have been previously studied in dolphins (1, 9, 14, 15, 29, 31), belugas (32), sea lions (9, 31), seals (9, 10, 31), killer whales (9, 31), polar bears (9), and sea otters (31). The study of these parameters in walruses is a new contribution of this work. Our group previously adapted another human kit for measuring phagocytosis in dolphins, namely the pHrodo Red *E. coli* BioParticles Kit (Thermo Fisher, Massachusetts, United States) (33). However, in most of these studies, the focus was on a methodological approach to the study of phagocytosis in these species and the detection of alterations in phagocyte function after *in vitro* exposure to environmental pollutants such as PCBs (31), heavy metals (14), tributylins (10), or silver nanoparticles (15). Only one of the previous studies aimed to generate a range of physiological values for phagocytic capacity and respiratory burst production in dolphins (29). Reif et al. (29) analyzed 40 wild dolphin blood samples and established a range of values for ingestion capacity and production of respiratory burst. On the one hand, they obtained the percentages of granulocytes and monocytes capable of ingesting the pathogen, in their case, a pre-labeled *Staphylococcus aureus*, with values of 19.9 ± 10.5% and 19.1 ± 10.1%, respectively. These percentages differ significantly from those described in this work (59.6 ± 1.27%). It’s important to note that Reif et al. (29) broke down the percentages by cell populations, while in this study, we present a general score of monocytes and granulocytes. Additionally, the protocols are not comparable. In their case, cells were incubated for 75 min with *Staphylococcus aureus*, while in our assay, they were incubated for 1 hour with *E. coli*. They used N-ethylmaleimide to block phagocytosis, while we used ice. The lysis of erythrocytes differs in both protocols, and they fixed the cells with 1% paraformaldehyde, a step omitted in our assay. Due to these differences in protocols, the results are not directly comparable. Hence, the standardization of the method provided by the adaptation of the phagocytic assay to marine mammals represents a significant advancement in this context. It’s also noteworthy that Reif’s et al. study (29) was conducted with a population of wild dolphins, while our group studied dolphins under human care. In their case, 89 wild dolphins were sampled, but only 40 samples were used for phagocytosis analysis. In our case, 15 dolphins have been sampled, but they have been tested periodically until reaching a total of 44 samples. While the number of samples is similar, our study takes a step further in elucidating the physiological values of dolphins’ phagocytic function. We obtained serial samples for 4 years from the same individuals, always ensuring they were in good health and excluding samples from sick animals. This rigorous approach has been consistently applied across all studied species. Although Reif’s et al. study (29) represents a significant advancement in understanding the immune function of wild dolphins, the challenging access to these animals limits them to one result per individual, making it difficult to ascertain whether the animals suffered from diseases during sampling. So obviously, the results are different between wild dolphins and dolphins under human care, but it is true that it is a very useful tool since most animals that arrive stranded on beaches are usually sick due to some infectious disease such as cetacean morbillivirus or *Brucella ceti*, both of which have been associated with immunosuppression (34). Indeed, co-infections of these microorganisms are common (34). However, the problem is that most animals that arrive stranded on our beaches are found dead, and in this case, there is no possibility of obtaining samples of fresh blood to conduct the study of immune function. This is what has happened in our study period; we have not been able to access fresh samples from stranded animals. However, we hope in the future to obtain samples and relate them to the study of possible infections in these animals. For beluga whales, walruses, sea lions, and seals, our study marks the first continuous examination of the same individuals in phagocytic function, establishing physiological values for each species. Interestingly, we have not observed significant differences in phagocytic function values between different species, suggesting a consistent action of phagocytes in marine mammals. While our study serves as an initial exploration in this field, it is crucial to expand the sample size, study more animals from different aquariums, and establish more robust results. Despite these considerations, the utility of our study is evident. Its successful application in various clinical cases of sick animals in aquariums, as demonstrated in the results section, highlights its value. This work presents two cases where the assay proved useful in detecting alterations in phagocyte function in animals suffering from infections, even before presenting symptoms. This underscores its effectiveness in enhancing preventive medicine in aquariums and better monitoring the health status of animals. Notably, the measurement of phagocytosis has never been used for diagnostic purposes in marine mammals before. In conclusion, the adaptation of the IngoFlow assay to these species emerges as a new diagnostic tool in aquariums. Furthermore, it holds potential for research studies, enabling result comparisons between different works conducted globally.

## Data availability statement

The original contributions presented in the study are included in the article/supplementary material, further inquiries can be directed to the corresponding authors.

## Ethics statement

The animal study was approved by Animal Care and Welfare Committee of the Oceanogràfic Aquarium (Reference: OCE-6-17). The study was conducted in accordance with the local legislation and institutional requirements.

## Author contributions

MF-B: Conceptualization, Formal analysis, Methodology, Writing – original draft, Writing – review & editing. AM-R: Conceptualization, Formal analysis, Visualization, Writing – review & editing. MV: Data curation, Writing – review & editing. CR-S: Data curation, Writing – review & editing. TÁ: Data curation, Writing – review & editing. DG-P: Conceptualization, Supervision, Validation, Writing – original draft, Writing – review & editing. CR-G: Conceptualization, Supervision, Writing – original draft, Writing – review & editing. J-EO’C: Conceptualization, Formal analysis, Investigation, Methodology, Supervision, Validation, Writing – original draft, Writing – review & editing.
